# The effects of line of sight on ground reaction force and pressure during landing

**DOI:** 10.1186/1757-1146-7-S1-A104

**Published:** 2014-04-08

**Authors:** Kyungock Yi

**Affiliations:** 1 Division of Human Movement Studies, College of Health Science, Ewha. Womans University

## 

“Keep your eye on the ball,” is an old sports adage. This is especially true in sports like volleyball, tennis, American football, and basketball. Among these sports, basketball is one of the few sports where airborne athletes cannot visually reestablish their relationship to the ground because they are focused on the ball. This typically happens when athletes are attempting to rebound a missed shot. Basketball players are expected to sacrifice their bodies in order to secure the ball, thus grabbing the ball is a greater priority than landing safely. As a result of this game-logic, athletes are unable to utilize their Vestibulo-Ocular Reflex (VOR) for landing. Does this create a higher risk for landing-related injuries? How does this altered line of sight affect landing strategies? What types of exercises can be implemented to minimize injury risk during these types of landings?

The purpose of this study was to evaluate the effects of line of sight on ground reaction force and pressure during landing. Subjects for this study were ten female university physical education majors. Subjects all jumped in bare feet to negate the effects of shoes. The independent variable for this study was line of sight during landing (forwards or upwards). Subjects focused on a vertical visual cue while jumping, and either continued to look upwards or changed their line of sight to a horizontal cue during landing. Dependent variables were ground reaction force (passive and active force via Kistler 9287BA, Switzerland) and pressure (area of center of pressure trajectory, path length, average velocity, forefoot and rear foot average force via the Zebris, Germany). Statistical analysis was performed using SPSS for the dependent t-test. Passive force variables (maximum passive force and number of passive force peaks) were significantly larger for subjects with an upward line of sight. Landing velocity for upward line of sight was also higher, although this difference was not statistically significant. In contrast there was no significant difference for active force regardless of line of sight.

For forward line of sight, there were significantly higher lengths for the x axis and path length. Subjects with an upwards line of sight had a significantly higher amount of forefoot force. These results show that different landing strategies are employed according to line of sight during landing.

Normally, athletes are able to adjust their line of sight while airborne in order to land safely. This mechanism was reflected in the forward line of sight. With a forward line of sight, subjects leaned forward during landing, utilizing greater hip flexion to cushion their impact. In addition, subjects made contact with both their toes and heels during landing, allowing the foot arch to flex and dissipate landing force. The greater use of the foot during landing resulted in greater x axis length and higher path length for forward line of sight.

In contrast, an upwards line of sight required a more upright spinal alignment, restricting hip movement when landing. This clearly illustrates the inter-relationship between the hip and neck during landing. As a result, subjects with an upward line of sight were far more dependent on flexion in the ankles to cushion their landing. Thus, subjects with an upward line of sight tended to land almost exclusively on their forefeet.

Athletes in jumping sports should practice landing with an upward line of sight in order to safely master this movement. Furthermore, in order to accommodate the forefoot landing strategy, basketball players should increase both their ankle and forefoot dorsi flexibility.

This study focused on line of sight and its influence on landing variables such as ground reaction force and center of pressure. However, dynamic balance incorporates many other variables including the visual, vestibular, and proprioceptive systems in addition to muscle strength, flexibility, and mobility. Future studies should investigate all of these variables in relation to landing with an upward line of sight in order to access which variable has the greatest influence on dynamic balance under these conditions. Furthermore, future studies should incorporate kinematic variables in addition to utilizing a downward line of sight.
